# Reduction of greenhouse gases emission through the use of tiletamine and zolazepam

**DOI:** 10.1038/s41598-022-13520-7

**Published:** 2022-06-09

**Authors:** Sonia Lachowska, Agnieszka Antończyk, Joanna Tunikowska, Martyna Godniak, Zdzisław Kiełbowicz

**Affiliations:** 1 Department and Clinic of Surgery, Faculty of Veterinary Medicine, Wroclaw University of Environment and Life Sciences, Pl. Grunwaldzki 51, 50-366, Wroclaw, Poland; 2 Royal (Dick) School of Veterinary Studies, The University of Edinburgh, Midlothian, EH25 9RG UK

**Keywords:** Ecology, Natural hazards, Health care, Health occupations, Medical research

## Abstract

Isoflurane is an anaesthetic gas widely used in both human and veterinary medicine. All currently used volatile anaesthetics are ozone-depleting halogenated compounds. The use of total intravenous anaesthesia (TIVA) allows to induce the effect of general anaesthesia by administering drugs only intravenously without the use of anaesthetic gases. This allows you to create a protocol that is safe not only for the patient, but also for doctors and the environment. However, so far, no anaesthetic protocol based on induction of anaesthesia with tiletamine-zolazepam without the need to maintain anaesthesia with anaesthetic gas has been developed. Our study showed that the use of this combination of drugs for induction does not require the use of additional isoflurane to maintain anaesthesia. Thanks to Dixon's up-and-down method we proved that with the induction of anaesthesia with tiletamine-zolazepam at a dose of 5 mg/kg the use of isoflurane is not needed to maintain anaesthesia in minimally invasive surgical procedures. Until now, this dose has been recommended by the producer for more diagnostic than surgical procedures or for induction of general anaesthesia. The maintenance was required with anaesthetic gas or administration of another dose of the tiletamine-zolazepam. The results obtained in this study will allow for a significant reduction in the consumption of isoflurane, a gas co-responsible for the deepening of the greenhouse effect, having a negative impact on patients and surgeons. These results are certainly the first step to achieving a well-balanced and safe TIVA-based anaesthetic protocol using tiletamine-zolazepam, the obvious goal of which will be to maximize both the safety of the patient, people involved in surgical procedures, and the environment itself. Being aware of the problem of the greenhouse effect, we are committed to reducing the consumption of anaesthetic gases by replacing them with infusion agents.

## Introduction

In recent years, people's awareness of Global Warming (GW) has increased. In 2021, the World Health Organization recognized climate change as the greatest threat to health in the world in the twenty-first century^1,2^. Rising Earth's temperature can lead to changes in rainfall patterns, a rise in sea level, and a wide range of impacts on plants, wildlife, and humans^3^. Gadani et al. even said that "Mankind may not be able to live through the twenty-first century if global warming and other forms of atmospheric pollution continue at the present rate." Therefore, there are many publications aimed at educating and deepening awareness in this direction, also of specific professional groups. This is to enable a multi-path reaction in various fields.

Global warming is linked to greenhouse gas emissions (GHG) such as: water vapor, carbon dioxide (CO_2_), methane (CH_4_), nitrous oxide (N_2_O), halogenated fluorocarbons (HCFCs), ozone (O_3_), perfluorinated carbons (PFCs), and hydrofluorocarbons (HFCs)^3–6^. They also include anaesthetic gases widely used in medicine, veterinary medicine, laboratories and research centers.

However, the involvement of these institutions in the exacerbation of GW has been largely ignored or justified under the guise of medical necessity^7^. It is worth mentioning that the impact of anaesthetic gases on the climate corresponds to about one-third of the climate impact of the use of electricity and heating networks^3^. However, the medical community does not want to ignore all this data for the welfare of current and future patients, and The American Society of Anesthesiologists has published a comprehensive document on what anaesthesiologists can do to make a "green" operating room^8^. Suggestions to reduce our carbon footprint included low-flow anaesthesia, the use of regional anaesthesia and total intravenous anaesthesia where possible, and less use of anaesthetic gases^9^. Andersen et al. stated that taking care to avoid the excessive use of anaesthetic gases has the double benefit of lowering health care and environmental costs.

As global citizens, scientists and veterinarians, we do not want to contribute to greenhouse gas (GHG) emissions. Thus, presented study shows a method of reduction of isoflurane, i.e. a greenhouse gas (GHG), for simple, minimally invasive procedures during the use of intravenous anaesthesia. In veterinary medicine, these types of minimally invasive and short procedures are very often performer under sedation or general anaesthesia. One of the most popular substances is propofol. However, the authors decided to abandon it due to the impact of unused propofol as waste on the environment and low analgesic effect^10–14^. Although other authors mention trace amounts of this drug, which are excreted unchanged by the patient, they do not take this into account^12,15^. The combination of tiletamine and zolazepam (TZ) are also used for induction and maintenance of anaesthesia. It is a medicine mostly used in dogs and cats however recent studies have assessed its validity for use in wild animals^16,17^. Zolazepam (benzodiazepine) was chosen to be combined with tiletamine (phencyclidine derivative) due to its anticonvulsant and muscle relaxant effects^18^. The volumes of TZ used in dogs are significantly smaller than the volumes of propofol, which already suggests less waste in the operating room. Additionally, according to the manufacturer: "No other adverse environmental effects (e.g., ozone depletion, photochemical ozone creation potential, endocrine disruption, global warming potential) are expected from this component". Another drug that the authors may have considered was ketamine, but due to its limited availability (in many countries it is necessary to have appropriate license for the use of this drug) it was decided not to use it.

The aim of the study is to evaluate the necessity of maintenance anaesthesia with isoflurane after induction with tiletamine-zolazepam in short and low invasive procedures in dogs. Therefore, this study proposes a universal and more environmentally friendly anaesthetic protocol.

## Material and methods

### Animals

After informed consent from the dog owners, 12 dogs were scheduled for the experimental procedure. Each patient had blood tests performed (haematology and biochemistry) and a clinical examination. Only ASA I–II dogs were enrolled in the study. The dogs weighed 16.5 ± 11.8 kg and were 3.3 ± 1.2 years old. Mixed breeds were the most frequent in this study.

Experiment has been approved by Local Ethical Committee for Animal Experiments at the Institute of Immunology and Experimental Therapy in Wrocław (No. 042/2020) and performed in accordance with relevant guidelines and regulations. The reporting in the manuscript follows the recommendations in the ARRIVE guidelines.

### Anaesthetic procedure

Prior to anaesthesia, a fasting period of 4–6 h was recommended, and water was withdrawn for 3 h prior to surgery. Each dog received light premedication with medetomidine-butorphanol (0.01 mg/kg, 0.1 mg/kg, respectively, Cepetor 1 mg/ml, CP-Pharma Handelsges. mbH Germany; Butomidor 10 mg/ml, Richter Pharma AG, Austria). 15 min after premedication, a 5-min pre-oxygenation was started, and a cannula (KD-FIX 22G or 24G) was inserted. The dogs were then transferred to the operating theatre and received fluid therapy with crystalloids (Optilyte, Fresenius Kabi Poland Sp. z o.o. Warsaw), at 10 mL/kg/h. They were positioned in lateral recumbency, and a heating mat was provided to maintain body temperature. Anaesthesia was induced intravenously with tiletamine-zolazepam (Zoletil 50 mg/ml, Virbac, France) at a dose of 5 mg/kg. Then dogs were then intubated and connected to anaesthetic equipment (Mindray Wato-Ex Pro 65 and Mindray BenVision N15, China). Anaesthesia was maintained with an anaesthetic gas-isoflurane (Iso-vet, Chanelle Pharma, Ireland). Fresh gas flow (FGF) with pure oxygen was set on high flow (153.8 ± 76.68 ml/kg/min) during first 15 min until the ISO-MAC calibrated to the desired level. Then the FGF was reduced to medium flow (96.29 ± 48.57 ml/kg/min). Throughout the experiment (from induction to patient stimulation time), hemodynamic and ventilation parameters were measured and recorded at 2-min intervals (heart rate (HR), respiratory rate (RR), non-invasive blood pressure (BP), saturation (SpO_2_), temperature (T), end-tidal CO_2_ (et-CO2), end-tidal isoflurane (et-ISO)).

### Experimental design

The examination time was divided into appropriate time points (T0–T7), in which the cardiovascular and respiratory parameters were measured. T0 was the first measurement taken immediately after induction of anaesthesia; T1 is the average of parameters taken in 14 and 16 min after induction of anaesthesia, when et-ISO had already been established at an appropriate level and the first stimulation was performed; T2–T7 are values taken every 2 min, which means that the last measurement (T7) was measured 10 min after stimulation.

The vaporizer was set at 0.7–0.0% vol. and the et-ISO adjusted for 15 min to the appropriate level determined by Dixon's Up-and-Down method (0.7–0.0 ± 0.1 et-ISO)^19^. Then, the level of anaesthesia was determined by performing stimulation. Positive or negative response to the noxious stimulus can only be assessed once in every patient^20,21^. In this study pressure on the footpad, phalanx, groin area and clenching the Backhaus on the skin were the noxious stimuli^22–25^.

The response to noxious stimulus was classified as positive if the patient did respond with movement (of the head, trunk or limbs) or if the HR, RR or BP raised by 20% compared to the baseline parameters before stimulation. The end-tidal isoflurane concentration (et-ISO) tested in three crossovers, in which the same patient could not appear twice, were used to calculate the MAC-ISO value^24,26^. The end-tidal isoflurane concentration in which the response was positive or negative, was recorded as the MAC value for the inhalant anaesthetic for each noxious modality for that patient^24^. In Dixon's Up-and-Down method the results are based on all the studied clinical patients^25,27,28^.

### Determination of MAC-ISO

End-tidal isoflurane concentration (et-ISO) was set to 0.7 vol.% in first patient^24–26^. It took up to 15 min to establish an appropriate level of anaesthetic. Following an et-ISO equilibration time the patient was given the noxious stimuli. If the response was negative, the et-ISO in the next patient was reduced by 0.1vol. %; if the response was positive, in the next dog et-ISO was increased by 0.1 vol. %. Changes in the response to the noxious stimulus between two consecutive dogs—a positive response followed by a negative response in the following dog or vice versa—were defined as ‘crossover’ values^25^.

### Dixon’s up-and-down method

According to Dixon's Up-and-Down method first step is to choose a series of test levels with equal spacing between doses. In our experiment the starting dose of the MAC isoflurane (MAC-ISO) was 0.7 and the spacing was 0.1^27^. The up-and-down method of Dixon is widely used for MAC determination not only in veterinary but also in human medicine^22,25,29^.

Next step is to carry out a series of trials following the rule of an increase in dose after a response is observed and a decrease in dose with no response present^27^. According to the rules of this method, the response to the noxious stimulus is only assessed once in each patient. The positive or negative response is then used to determine the MAC of the inhalation anaesthetic for the subsequent patient^22^. In the present study, the noxious stimulus consisted of compressions of paw pad, phalange, groin area and skin pinching using Backhaus clamps^30–32^.

In this method we look for the crossovers which are defined as the opposite response to the stimulation in two consecutive patients (positive and then negative or vice versa). To increase the reliability of the results, the response to noxious stimulus and the MAC level of isoflurane 0.0 was checked 3 times.

### Statistical analysis

Statistical analysis included descriptive statistics and normality testing using the Kolmogorov–Smirnov. Repeated measurement analysis of variance was applied to compare the vital parameters at different time points.

## Results

**Figure 1:**
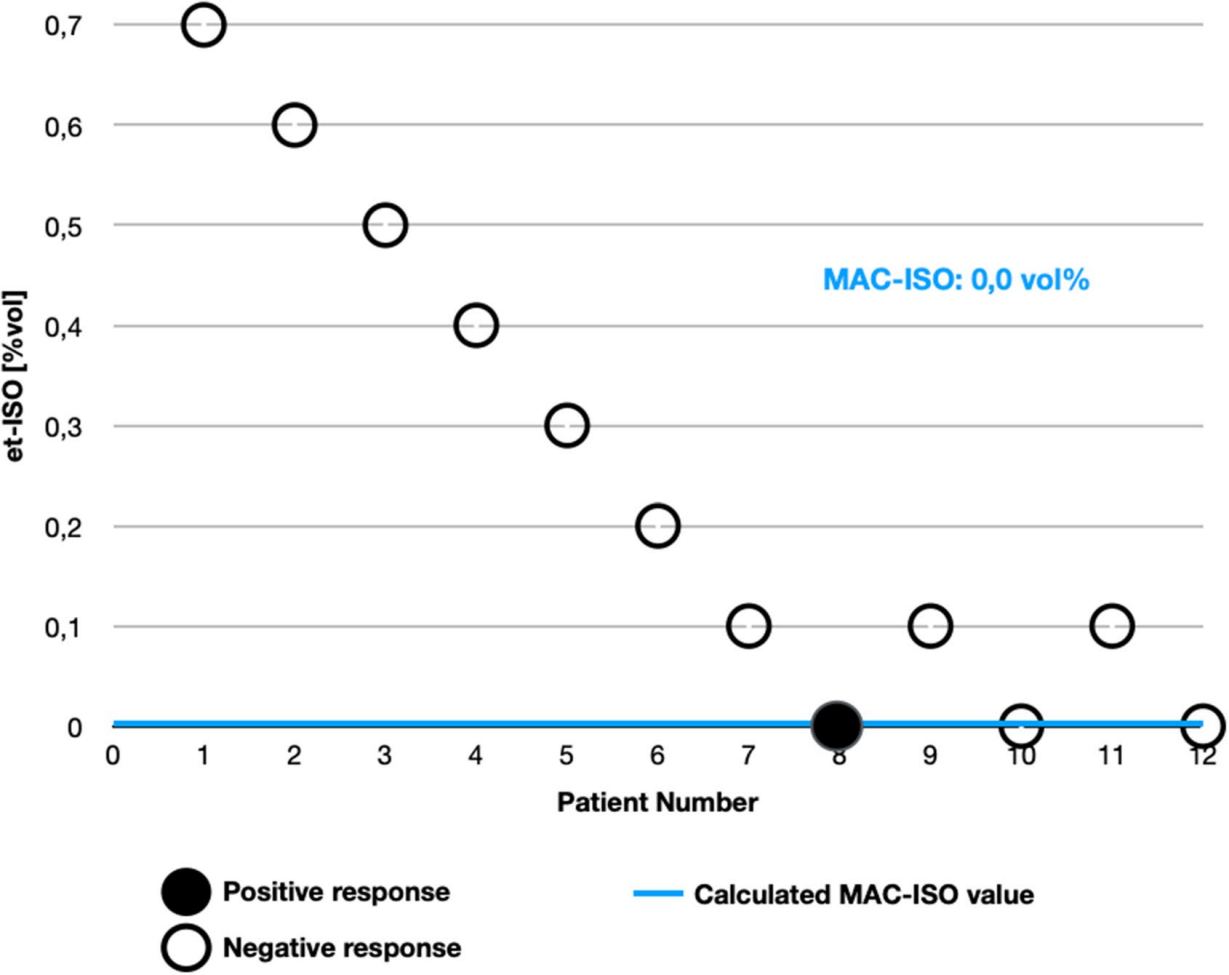
Results of using the Dixon up-and-down method.

The administration of tiletamine-zolazepam resulted in the reduction of MAC-ISO to 0.0 vol.% (Fig. 1).

**Figure 2:**
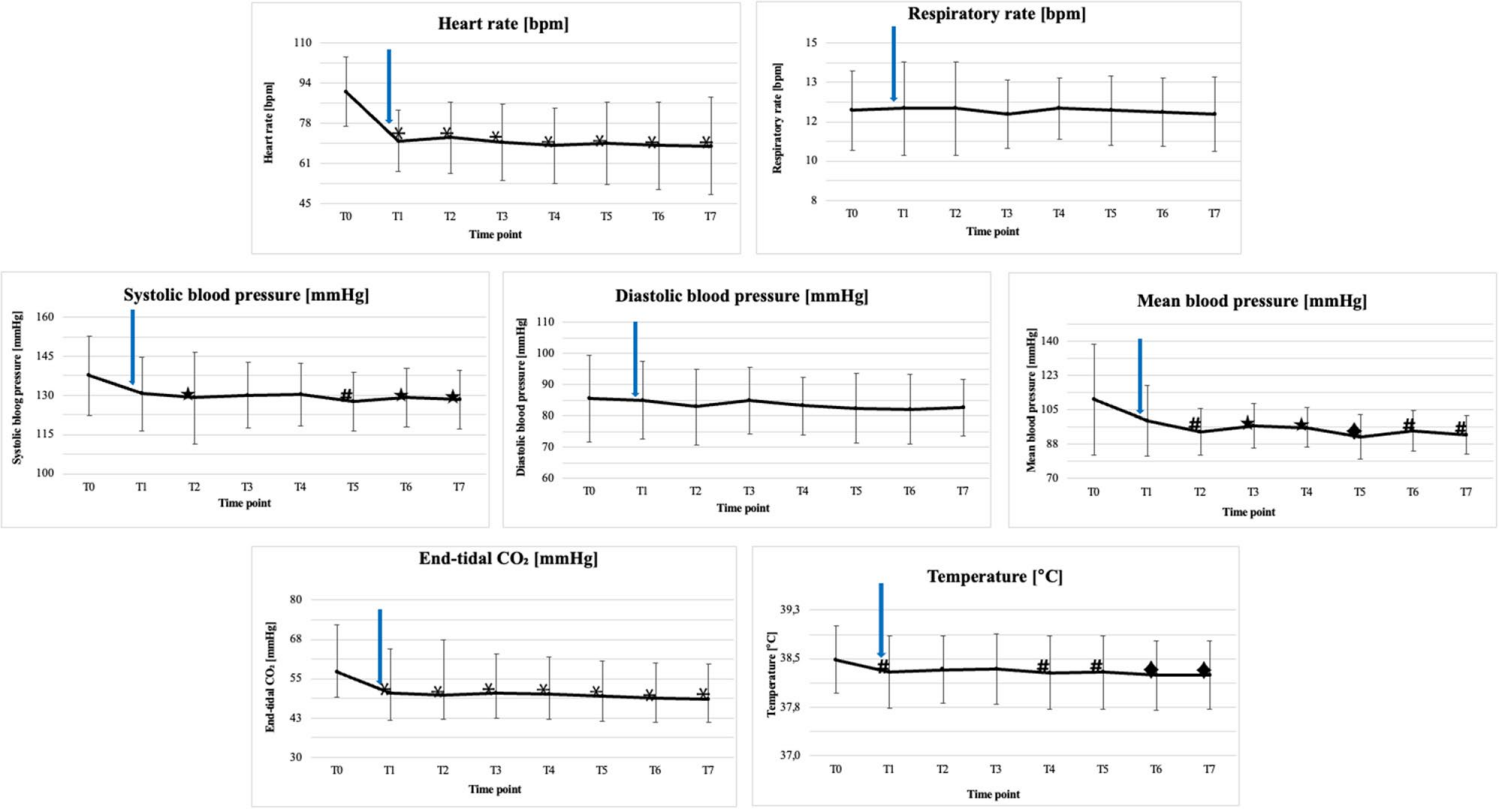
Graph of the mean values of the measurements (HR, RR, SYS, DIA, MEAN, et-CO_2_, T) at the respective time points. Statistically significant differences between T0 and the corresponding time point have been marked according to following rules: filled star for *p* ∈ (0.05, 0.01 >; number sign for *p* ∈ (0.01, 0.001 >; filled diamond for *p* ∈ (0.001, 0.0001 >; asterisk for *p* ∈ (0.0001, 0.00001 >; The blue arrow shows when stimulation started.

The statistical analysis revealed differences in HR between T0 and all subsequent time points (Fig. 2).

No significant differences were found in the measurements of respiratory rate (*p* = 0.986).

The blood pressure test showed statistically significant differences for systolic (*p* = 0.01) and mean (*p* = 0.0005) blood pressure, but no such significance for diastolic pressure (*p* = 0.46) was shown. Systolic blood pressure decreased in comparing T0 to T1, remaining constant to T4, then decreased slightly in T5. Mean blood pressure decreased significantly over the next 3 time points, keeping its value down to T4. There was a slight decrease in mean blood pressure in T5 which was then sustained until the end of the study (Fig. 2).

End-tidal carbon dioxide showed statistically significant differences (*p* < 0.000001). There were significant statistical differences between T0 and T1–T7 (Fig. 2).

Statistical significances were found during temperature measurements (*p* = 0.00011). The temperature decreased between T0 and T1 and then gradually dropped from T4 to the end of the procedure (Fig. 2).

## Discussion

The knowledge about the influence of anaesthetic gases is evolving and deepening from year to year, which was propagated by many authors^3,7,14,33,34^. In 2011 Ishizawa et al.^35^ said that "All volatile anaesthetics that are currently used are halogenated compounds destructive to the ozone layer". However, nearly a year later, Andersen et al. clarified some misconceptions or errors in their study, and proved that isoflurane is among them, the only one containing chlorine in its structure, which contributes to the catalytic destruction of stratospheric ozone. In contrast, Caycedo-Marulanda et al. suggested that anaesthetists should abandon the use of anaesthetic gases, if possible, replacing them with a different strategy—intravenous or regional anaesthesia, which may have reduced the carbon footprint. The possibility of replacing nitrous oxide in the anaesthetic gas mixture was also considered to reduce the harmful effects on global warming. However, after replacing the carrier gas with an air/oxygen mixture, this effect turned out to be even greater^3,7^.

During anaesthesia with anaesthetic gas, the patient metabolizes less than 5% of the total delivered anaesthetic, the vast majority, is routinely discharged into the atmosphere through the operating room (OR) cleaning system, if there is one^3^. The medical waste gases are usually vented out of the building as medical waste gases, and most of the organic anesthetic gases remain for a long time in the atmosphere where they have the potential to act as greenhouse gases^36^. In addition, it is worth mentioning waste anaesthetic gases (WAGs), i.e., small amounts of inhaled anaesthetics present mainly in the operating room and post-anaesthesia care unit ambient air, which include isoflurane, sevoflurane, desflurane and nitrous oxide^37,38^. The operating environment is polluted by WAG mainly due to anaesthetic techniques, anaesthesia workstation, and OR with or without a scavenging system^10^. Research conducted at an American academic medical center has shown that anaesthetic gases are responsible for over 50% of the carbon footprint of operating rooms^39,40^. Thus, it is not without reason that anaesthesiology is said to play a significant role in loading GHG^41,42^.

In many countries there are limits of occupational exposure to WAGs, but they are rarely met, especially in small veterinary clinics. It is also worth mentioning the possible effects of anaesthetic gases on staff involved in the surgery—such as headache, irritability, fatigue, nausea, dizziness, difficulty judgment, coordination, and even more serious changes such as kidney and liver damage and neurodegenerative conditions^37,38,43,44^. Additionally, Sherma et al. acknowledge that WAG is mutagenic and teratogenic, and suggest the use of a total intravenous anaesthetic (TIVA) when there is a known pregnant provider in the operating arena^45^.

Reducing the amount of anaesthetic gas through the use of low-flow anesthesia is an obvious way to reduce the emission of these gases into the environment^46–48^. However, it is worth mentioning that in veterinary medicine the use of low-flow anaesthesia is not commonly used mainly due to the lack of appropriate equipment and monitoring. This equipment is very expensive but also critical to monitoring the inspired oxygen concentration and to avoiding hypoxic mixtures when using low FGF^48–50^. Additionally, it is difficult to apply the low flow anaesthesia principle with short procedures. It is recommended to use a high gas flow until the MAC stabilizes and then the gases should be lowered to low values^46^.

In this study the MAC-ISO values were obtained by average of 3 crossovers rounding the result to a decimal place. Paul et al. find that the more crossovers, the more reliable the results, however the improvement diminishes when the number of crossovers exceeds six^28^. In turn, Dixon suggests that the experiment should be continued until the number of intersections is four. In our study, the number of intersections is three due to the focuses of results at the MAC value of 0.0%^27^. It was considered that further experiment could lead to a larger inter-individual variability led to more "outlier" estimates for MAC^28^.

The authors of other publications present studies in which the use of isoflurane in induction with tiletamine-zolazepam is very high. MAC-ISO values are between 1.0 and 1.6, and the vaporizer is set to high values of 1.5–2.5 vol.%^51–53^. Due to the lack of information on inducing an appropriate level of anaesthesia in dogs using TZ induction and its maintenance with isoflurane, it was decided to consider the pig study by Malavasi et al.^54,55^. Authors demonstrated that the tiletamine-zolazepam in intramuscularly administration significantly reduced the MAC-ISO. In literature the anaesthetic protocols for dogs are based on high doses of isoflurane during use of tiletamine-zolazepam in different administration route also in multimodal anaesthesia^52,53,56^. Due to the risk of less accurate average MAC estimate and use of insufficient number of individuals needed to attain a desired number of crossovers authors decided to consider as a starting MAC value 0.7 vol. %. The average MAC estimate typically increases while starting with the initial concentrations larger than population MAC^28^.

Additionally, it is worth mentioning the importance of the light premedication used in the following experiment in conjunction with the induction of anaesthesia in the form of a low dose of tiletamine and zolazepam. In the available literature, combinations of opioids with an alpha 2 agonist are used for premedication, but at higher doses^57,58^. Those anaesthetic protocols are not applicable to all patients.

In most studies, the noxious stimulus was tail clamp^25,59^. In our research, the stimulation was more advanced and included many more types of stimuli (pressure on the footpad, phalanx, groin area and clenching the Backhaus on the skin). The result was a more in-depth and reliable assessment of the level of sedation and analgesia in the studied patients.

## Conclusion

All of the above arguments highlight the need to create anaesthetic protocols which would allow the limitation or even elimination of the use of anaesthetic gases. This study proved that patients undergoing light premedication and induction of anaesthesia with tiletamine-zolazepam at a dose of 5 mg/kg without the use of isoflurane did not respond to minimally invasive stimuli. Therefore, authors hypothesized that short and less-invasive procedures do not require anaesthetic gases contributing to reduction of the GHG emission.

## Data Availability

The authors declare that all other data supporting the findings of this study are available within the paper.
